# Supplementary figures and images for: The relationship of mRNA with protein expression in CD8+ T cells associates with gene class and gene characteristics
Source: PLoS One. 2022 Oct 19;17(10):e0276294. doi: 10.1371/journal.pone.0276294 (PMC9581405; doi:10.1371/journal.pone.0276294)

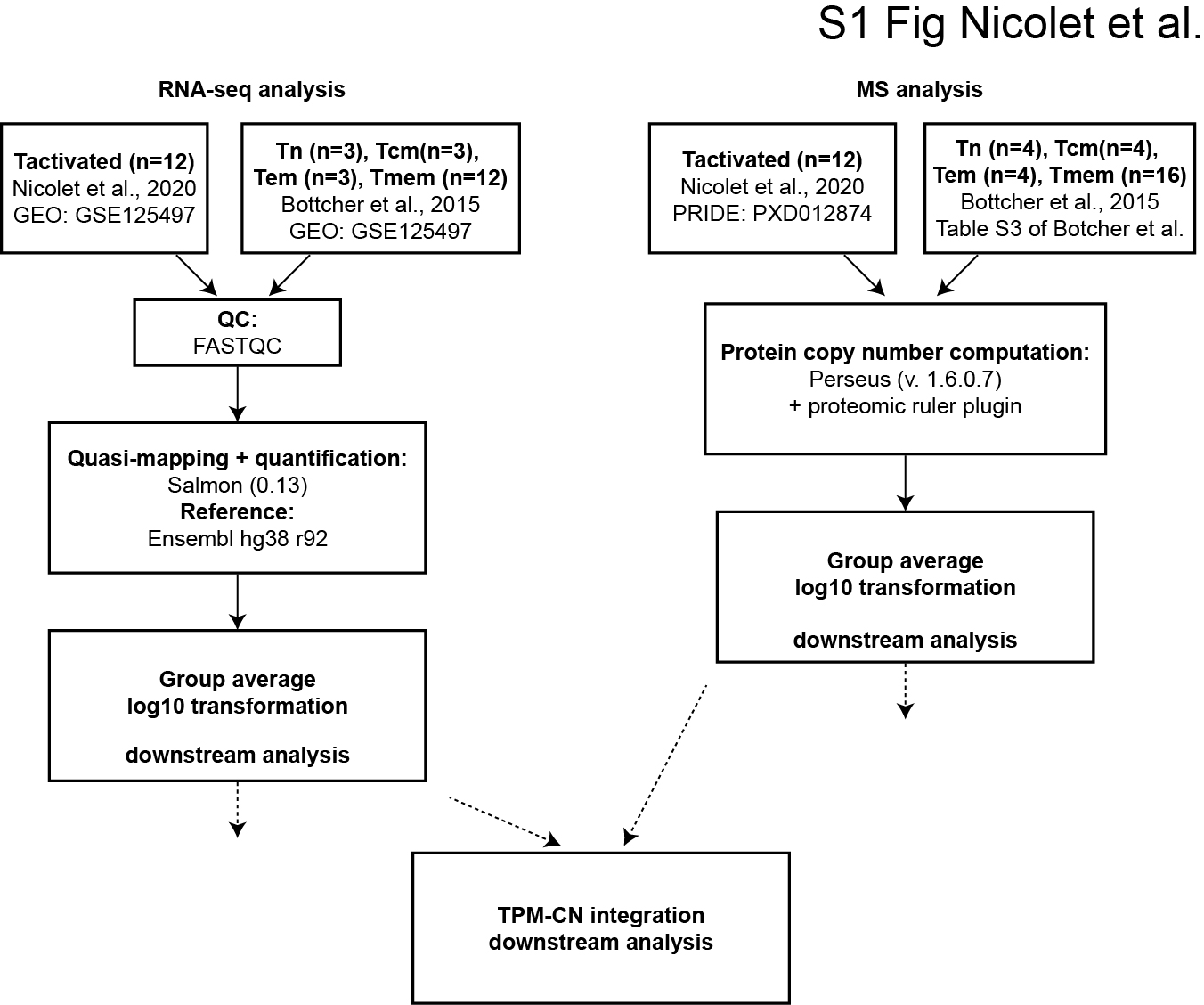

Supplement: S1 Fig — Workflow of the analysis of RNA-sequencing (RNA-seq) and Mass spectrometry (MS) data of blood-derived naïve (TN; CD45RA+ CD62Lhigh; n = 3), central memory (TCM; CD45RO+ CD62Lhigh CX3CR1-; n = 3), effector-memory (TEM; CD45RO+, CD62Llow, CX3CR1high; n = 4) and memory CD45RO+ CD8+ T cells (TMEM; n = 16) CD8+ T cell subsets and of CD8+ T cells that were activated for 2 days with αCD3/αCD28, cultured for 4 days, and were then re-activated for 4h with PMA-Ionomycin (TActivated; n = 12; from [23]). (JPG) [file pone.0276294.s001.jpg]

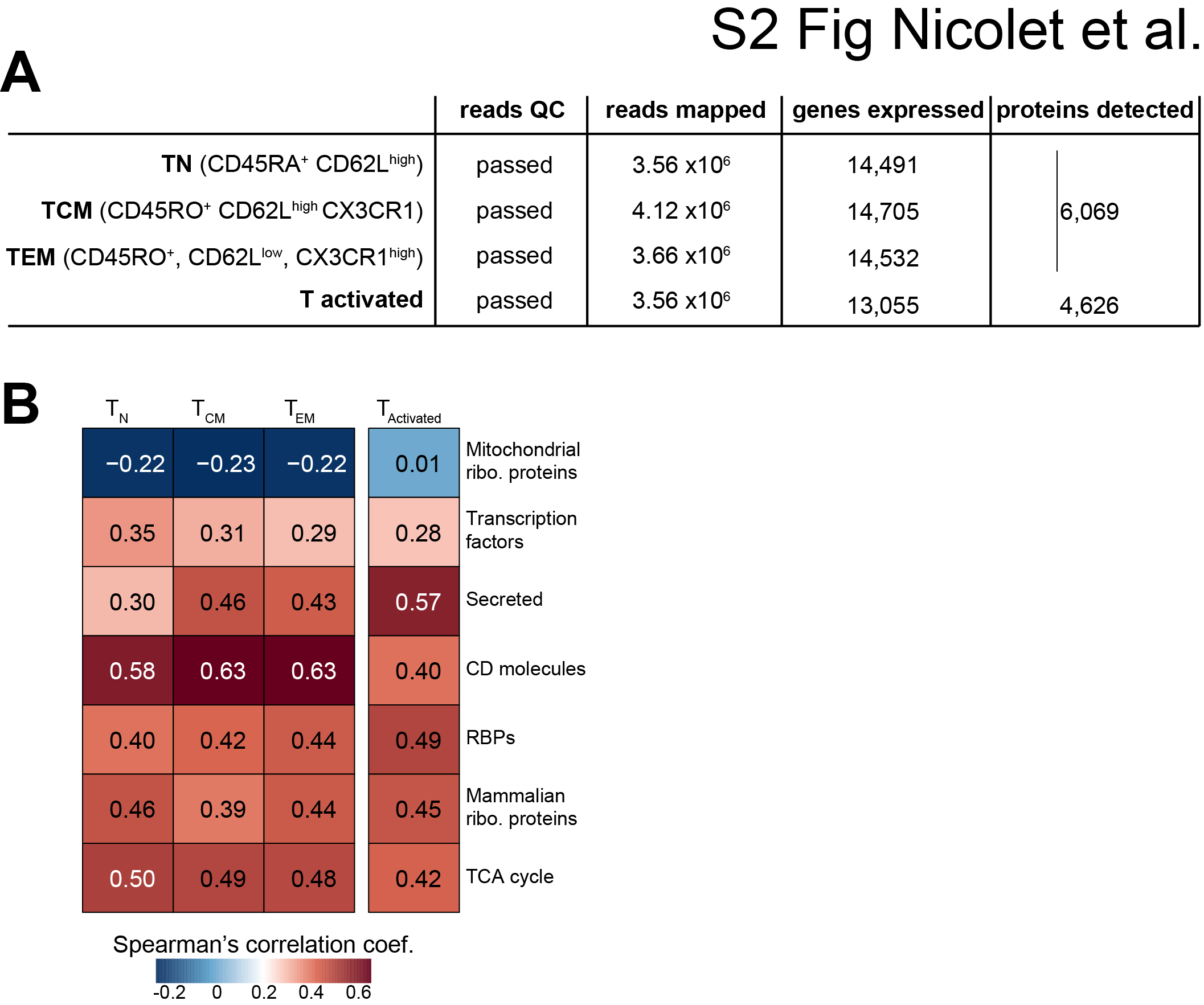

Supplement: S2 Fig — (A) Quality control (QC) using FASTQC, number of reads mapped to transcriptome, number of gene expressed and protein detected for blood-derived, naïve (TN; CD45RA+ CD62Lhigh; n = 3), central memory (TCM; CD45RO+ CD62Lhigh CX3CR1-; n = 3) and effector-memory (TEM; CD45RO+, CD62Llow, CX3CR1high; n = 4) CD8+ T cell subsets and for CD8+ T cells that were activated for 2 days with αCD3/αCD28, cultured for 4 days, and were then re-activated for 4h with PMA-Ionomycin (TActivated; n = 12; from [23]). (B) Spearman’s correlation of the integrated mRNA and protein abundance of gene classes indicated in Fig 2 during T cell differentiation and upon T cell activation. (JPG) [file pone.0276294.s002.jpg]

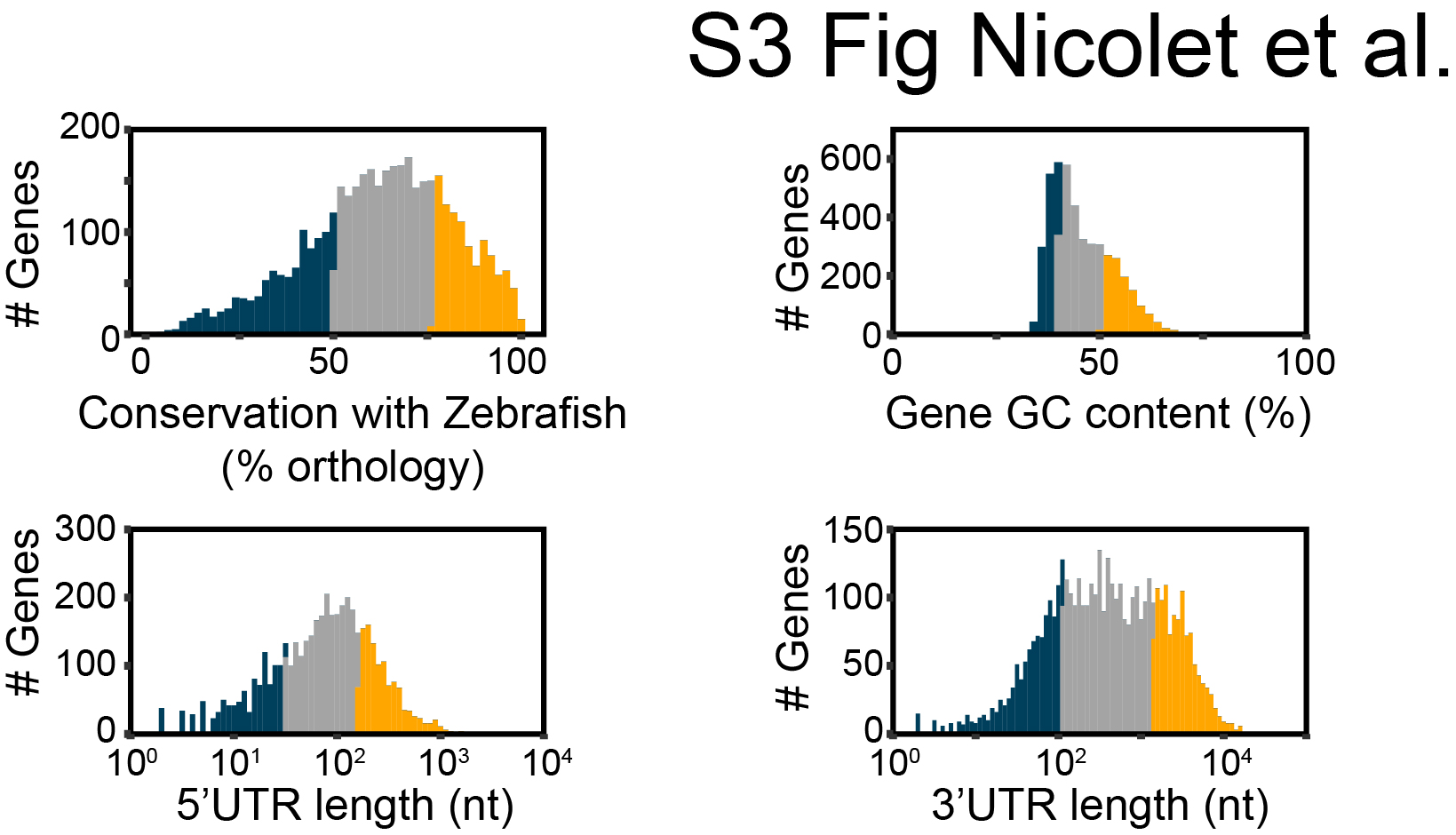

Supplement: S3 Fig — Distribution and color-coded cut-off (top 25%; mid. 50%; bottom 25% of all genes) for stratifying orthologous gene sequence conservation between Human and Zebrafish (in %), GC content (in %), 5’ and 3’UTR length (in nucleotides; nt). Only gene products detected at mRNA and protein levels are shown. (JPG) [file pone.0276294.s003.jpg]

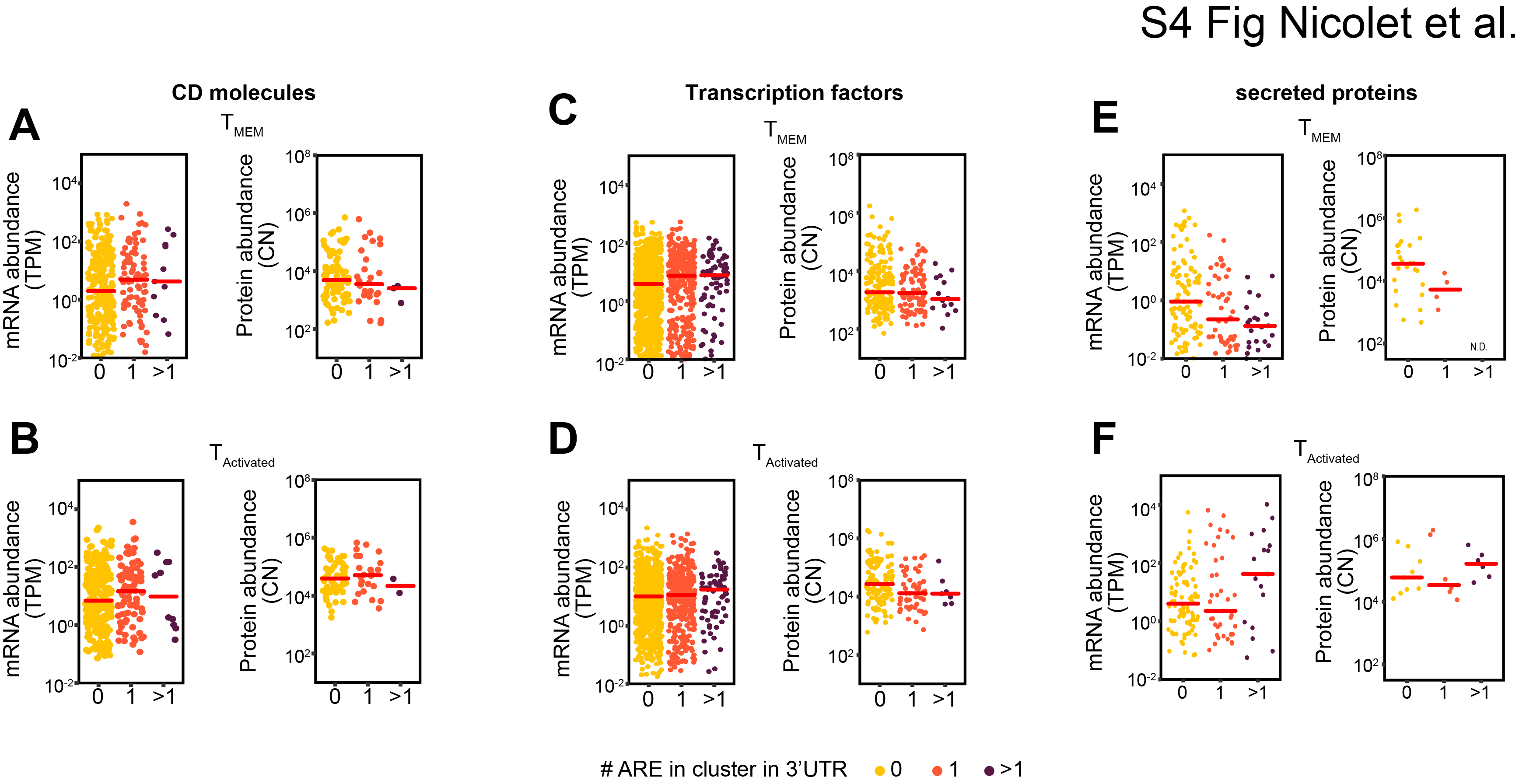

Supplement: S4 Fig — (A-F) mRNA (TPM) and protein (CN) abundance in log10 space for CD molecules (A-B), transcription factors (C-D), or secreted molecules (E-F) in TMEM (A, C, E) or TActivated CD8+ T cells (B, D, F). Genes products are color-coded according to the number of ARE clusters (ARED annotation) within the 3’UTR. Differences were assessed with a two-tailed t-test followed by adjustment of p-value using the Benjamini-Hochberg procedure. N.D.: not detected. (JPG) [file pone.0276294.s004.jpg]
