# Supplementary material for: The relationship of mRNA with protein expression in CD8+ T cells associates with gene class and gene characteristics
Source: PLoS One. 2022 Oct 19;17(10):e0276294. doi: 10.1371/journal.pone.0276294 (PMC9581405; doi:10.1371/journal.pone.0276294)
Supplement: Attachment — Submitted filename: PONE-D-22-19814_ReviewerComments.pdf [file pone.0276294.s007.pdf]

The study integrated gene expression with corresponding protein levels and compared the gene-protein correlations across different T cell differentiation/activation status. As previously seen by other studies, the authors show low gene-protein correlations; these were independent of differentiation status. They further show that the mRNA -protein expression relationship was gene class specific and is associated with particular sequence characteristics.

The manuscript is generally well written and easy to understand. A few interesting links are shown that could provide insight into the low gene-protein correlation commonly seen. However, some aspects of the work need clarification and listed below.

1. Methods, lines 88 – 90: Give the number of biological samples in each group. Even though it's given in Fig.S1, this information should be provided early and in methods.
2. Lines 96 -97: state the number of biological replicates in each group
3. Methods: The authors have averaged across biological replicates, which could potentially dilute the expression signal due to variability between samples - how did they check low variability between replicates before merging? Did the authors compare the correlation across replicates within each differentiation state before merging?
4. Methods, Line 101 – it is unclear what quality control approach/metrics the authors are referring to here. Is this based on FASTQC?
5. Line 133 – check if it should read "bottom 25%" as opposed to "top 25%"
6. Results - A study/analysis workflow diagram with the number of samples in each group will be helpful for the reader to follow.
7. Line 161, "average 47.35 pg of protein per cell (n=16; Fig 1C)" – please check the "n=16" does not match the numbers given in the figure legend and the samples denoted in the bar graph.
8. Line 170 – are these correlations statistically significant? Provide p-values.
9. Line 165-173: Provide P-values for the correlation and regression analysis.
10. Lines 173 – 174: "no effect on the correlation and slope despite the cell mass per cell nearly doubled". Have the authors considered ranking the proteins and genes and looking at the overlap of the top ranked genes/proteins. Are these genes enriched in a particular pathway or gene ontology function?
11. Lines 178-204, what was the choice of using these gene classes? Did the authors consider looking at the gene-protein correlation between the core gene sets identified by Böttcher et al. and Nicolet et al. and how those changes across the differentiation stages?
12. Figure 2: Do the authors also get similar values when using Spearman's correlation? Even though Pearson's correlation does not assume normality, it can be sensitive to outliers – instances where few gene are very highly expressed.
13. Figure 2: The Ribosomal Protein panel is showing correlation calculated across groups (dark green and light green)– indicate in the legend what those are
14. Figure 2 B-C: A suggestion – change the label on the colour key to be "Pearson correlation coefficient" for (B) and "Beta coefficient" for (C). Are all the values shown in the heatmap statistically significant? Specify where significant.
15. Figure 4 A,C,E,G and Figure 5A,B - a boxplot (log2 or log10 transformed values) might have been a better representation. Specify in the legend what the red horizontal lines indicate. Also, did the authors explore the option of plotting gene-protein ratio. Give P-values for all regression analysis.
    For all the regression analysis, p-values should also be provided.
    Lines 287 – 298 – within each gene class, the genes were first sub-setted based on the number of ARE, and then the regression analysis was run. From the figures, it seems like regression was run with less than 10 genes. Why did the authors not run a multivariable regression analysis on all genes within the gene class and adjust for

the number of ARE in the model? The slope/p-values of the multivariable regression should be compared with that univariate regression slope .

16. Inconsistent order of Figure labelling check Figures 3,4,5. Suggestion: Label top right panel as B and bottom left panel as C.
17. Figure 5B – What does n.s stand for?
18. Supplementary Figure 3 – since non-transformed values are plotted, the mean would be driven by extreme values. E.g., Supp Fig 3B (group >1)
19. The authors should also mention in the discussion the role of *Trans*-acting DNA variants (eQTLs/pQTLs) may specifically affect mRNA or protein levels of genes which could also explain the low correlation seen.
