# Supplementary material for: The relationship of mRNA with protein expression in CD8+ T cells associates with gene class and gene characteristics
Source: PLoS One. 2022 Oct 19;17(10):e0276294. doi: 10.1371/journal.pone.0276294 (PMC9581405; doi:10.1371/journal.pone.0276294)
Supplement: Attachment — Submitted filename: Response to reviewers_Nicolet&Wolkers_PONE-D-22-19814.docx [file pone.0276294.s008.docx]

**Point-to-point response for PONE-D-22-19814: *The relationship of mRNA with protein expression in CD8+ T cells associates with gene class and gene characteristics* by Benoit and Wolkers.**

**Editorial office:**

1. ***Please ensure that your manuscript meets PLOS ONE's style requirements, including those for file naming.***

Confirmed

2. ***We note that the grant information you provided in the ‘Funding Information’ and ‘Financial Disclosure’ sections do not match. When you resubmit, please ensure that you provide the correct grant numbers for the awards you received for your study in the ‘Funding Information’ section.***

Please be advised that the Oncode Institute does not provide a grant number.

3. ***Thank you for stating the following in the Funding Section of your manuscript: "This research was supported by the Oncode Institute, and the European research council (ERC-Printers 817533)."We note that you have provided funding information that is not currently declared in your Funding Statement. However, funding information should not appear in the Acknowledgments section or other areas of your manuscript. We will only publish funding information present in the Funding Statement section of the online submission form.
   Please remove any funding-related text from the manuscript and let us know how you would like to update your Funding Statement. Currently, your Funding Statement reads as follows: "Funded study NO - The funders had no role in study design, data collection and analysis, decision to publish, or preparation of the manuscript" Please include your amended statements within your cover letter; we will change the online submission form on your behalf.***

Thank you for noticing this mismatch. Our study was supported by the Oncode Institute (grant to M. Wolkers), and the European research council (ERC-Printers 817533). These funding bodies had no role in study design, data collection and analysis, decision to publish, or preparation of the manuscript. We hope this clarifies our statement.

4. ***We note that you have stated that you will provide repository information for your data at acceptance. Should your manuscript be accepted for publication, we will hold it until you provide the relevant accession numbers or DOIs necessary to access your data. If you wish to make changes to your Data Availability statement, please describe these changes in your cover letter and we will update your Data Availability statement to reflect the information you provide.***

We apologize to have crossed the wrong box during submission. Please be advised that all sources are already accessible: the datasets analyzed were already previously published, and are referred to in the methods section, including their accession numbers. We did not generate new data. The analyzed data are presented in supplemental tables 1+2 and the scripts to reproduce our analysis on github : <https://github.com/BenNicolet/mRNA_and_protein_intregration_in_CD8_Tcells>.

5. ***Please include a copy of Supplemental table 3 which you refer to in your text on page 5.***

We apologize for this confusion: On Line 88 of page 5, we referred to Supplemental table 3 of *Bottcher et al.* (reference 22). To clarify, we now state “Supplemental table 3 of reference [22]”.

6. ***Please review your reference list to ensure that it is complete and correct. If you have cited papers that have been retracted, please include the rationale for doing so in the manuscript text, or remove these references and replace them with relevant current references. Any changes to the reference list should be mentioned in the rebuttal letter that accompanies your revised manuscript. If you need to cite a retracted article, indicate the article’s retracted status in the References list and also include a citation and full reference for the retraction notice.***

We have adjusted the reference list. Of note, our citation manager did not flag any of our references as retracted (Retraction Watch database).

**Reviewers' comments:**

**Reviewer #1:**

***In “The relationship of mRNA with protein expression in CD8+ T cells associates with gene class and gene characteristics”, Nicolet et. al performed bioinformatic analyses of previously published RNA and protein data from primary human CD8+ T cells. They demonstrate, as has been reported in other contexts, poor correlation between RNA and proteins levels. They furthered their analyses by demonstrating unique correlation patterns between different classes of genes. Overall, this study is scientifically sound, and despite being almost purely in silico, it represents useful data that should be published and available to the field. My concerns are relatively minor, mostly aimed at ensuring clarity in the results:***

We thank the reviewer for their time and appreciation of our work.

1. ***The activated cells were treated with Monensin. Can the authors present data or citations as to whether Monensin impacts protein and/or RNA levels?***

We thank the reviewer for this important comment. We have previously tested the effect of monensin on mRNA and protein level of IFNg using Flow-FISH ([doi.org/10.4049/jimmunol.1601531](https://doi.org/10.4049/jimmunol.1601531)). Monensin resulted in little to no decrease in mRNA staining (Figure 4 of the reference above). In contrast, brefeldin A affected the mRNA levels (data not shown in the original paper). To our knowledge, no study has yet assessed the proteome/transcriptome-wide effect of monensin (or brefeldin A) treatment. We highlight this point on line 211.

2. ***In figure 4 the authors examine RNA/protein levels when stratifying gene based on various characteristics, such as conservation or UTR length. In Fig 3 they show that genes within each class can have wide ranges for these values. If the authors performed a similar analysis as Fig 4, except grouping the data by gene class, are the trends reported in Figure 4 consistent between each class?***

We thank the reviewer for this interesting comment. We have provided such analysis for AU-rich elements in Figure 5-6 & S3. Because most gene classes do not contain sufficient numbers of gene/proteins (due to detection level in MS) to allow for a reliable analysis using quartile (top 25%/ mid 50% / bottom 25%), similar analysis for GC content, and UTR length of all gene classes is challenging. We therefore refrain from running such analysis to prevent potentially misleading results due to small group size.

**Reviewer #2:**

***The authors have not thought much on the appropriate statistical analysis/test to run, in particular with the regression analysis. Data could have been transformed and presented better. For example, using box plots. See the attached document for comprehensive comments on the manuscript.***

***The study integrated gene expression with corresponding protein levels and compared the gene-protein correlations across different T cell differentiation/activation status. As previously seen by other studies, the authors show low gene-protein correlations; these were independent of differentiation status. They further show that the mRNA -protein expression relationship was gene class specific and is associated with particular sequence characteristics.***

***The manuscript is generally well written and easy to understand. A few interesting links are shown that could provide insight into the low gene-protein correlation commonly seen. However, some aspects of the work need clarification and listed below.***

We thank the reviewer for their time and appreciation of our work.

1. ***Methods, lines 88 – 90: Give the number of biological samples in each group. Even though it’s given in Fig.S1, this information should be provided early and in methods.***

We now added the number of biological replicates.

2. ***Lines 96 -97: state the number of biological replicates in each group***

Now included.

3. ***Methods: The authors have averaged across biological replicates, which could potentially dilute the expression signal due to variability between samples - how did they check low variability between replicates before merging? Did the authors compare the correlation across replicates within each differentiation state before merging?***

Sample-to-sample variation had been checked in the respective original publications. It was limited between replicates, as exemplified by PCA and correlation plots.

The rationale to average samples of a group is to remove biological variation. The resulting averaged measurements are more representative than measurements of single samples. In addition, the averaging should not cause any over- or underestimation of the mRNA/protein relation, as outliers will regress to the mean. This is desirable in our analysis as not to bias the results. We have now included this information (line 90, 93).

4. ***Methods, Line 101 – it is unclear what quality control approach/metrics the authors are referring to here. Is this based on FASTQC?***

To clarify our approach, we now state: “*All samples were analyzed with FASTQC (Babraham institute: https://www.bioinformatics.babraham.ac.uk/projects/fastqc/) and passed our quality control*”.

5. ***Line 133 – check if it should read “bottom 25%” as opposed to “top 25%”***

We thank the reviewer for noticing this mistake, and we corrected it.

6. ***Results - A study/analysis workflow diagram with the number of samples in each group will be helpful for the reader to follow.***

We thank the reviewer for this suggestion. We now included an analysis workflow (NEW Fig S1).

7. **Line 161, “average 47.35 pg of protein per cell (n=16; Fig 1C)” – please check the “n=16” does not match the numbers given in the figure legend and the samples denoted in the bar graph.**

We thank the reviewer for noticing this mistake. Figure legend and line 161 were indeed erroneous. We now indicated the correct number of replicates, and in NEW Fig S1.

8. ***Line 170 – are these correlations statistically significant? Provide p-values.***

We tested for significance of both correlation (Pearson’s and Spearman’s) and regression and report a p-value of <2e-16 for both. We now indicate in the methods section: “To test for statistical significance of regressions, we used an F-test. To test for statistical significance of correlation, we used cor.test() of the R {stats} package.”.

9. **Line 165-173: Provide P-values for the correlation and regression analysis.**

Please see our answer to point 8.

10. ***Lines 173 – 174: “no effect on the correlation and slope despite the cell mass per cell nearly doubled”. Have the authors considered ranking the proteins and genes and looking at the overlap of the top ranked genes/proteins. Are these genes enriched in a particular pathway or gene ontology function?***

We thank the reviewer for this comment. We did not include this analysis as previous work has performed this, e.g. *doi.org/10.1016/j.immuni.2018.04.008; or doi.org/10.1038/ni.3314.*

11. ***Lines 178-204, what was the choice of using these gene classes? Did the authors consider looking at the gene-protein correlation between the core gene sets identified by Böttcher et al. and Nicolet et al. and how those changes across the differentiation stages?***

Gene classes were chosen based on protein classes found in the human protein atlas (<https://www.proteinatlas.org/humanproteome/proteinclasses>). The correlation of core gene sets is a good suggestion, yet we find that it would distract the reader from the core message of this paper.

12. ***Figure 2: Do the authors also get similar values when using Spearman’s correlation? Even though Pearson’s correlation does not assume normality, it can be sensitive to outliers – instances where few gene are very highly expressed.***

We have used the Spearman’s correlation coefficient in Fig S2B, and indeed find similar patterns. We now include this information in the manuscript. On lines 241-242, it now reads “Similar patterns were observed with Spearman’s correlation coefficient and with the mRNA-to-protein slope for gene classes (Figs 2C, S2B).”.

13. ***Figure 2: The Ribosomal Protein panel is showing correlation calculated across groups (dark green and light green)– indicate in the legend what those are***

To clarify this, we now state: “*The correlation was performed independently for mammalian (light green) and mitochondrial (dark green) ribosomal proteins*” in the legend of Figure 2.

14. **Figure 2 B-C: A suggestion – change the label on the colour key to be “Pearson correlation coefficient” for (B) and “Beta coefficient” for (C). Are all the values shown in the heatmap statistically significant? Specify where significant.**

We thank the reviewer for this suggestion. We now added “slope (also known as Beta-coefficient)“ to the figure legend, and coded the correlation/regression that returned an F-test p-value <0.05 with a “*” in the heatmaps of figure 2. Accordingly, we added “*To test for statistical significance of regressions, we used an F-test.”* to the method section (line 144).

15. ***Figure 4 A,C,E,G and Figure 5A,B –*** A) ***a boxplot (log2 or log10 transformed values) might have been a better representation.*** B) ***Specify in the legend what the red horizontal lines indicate. Also, did the authors explore the option of plotting gene-protein ratio.*** C*)* ***Give P- values for all regression analysis. For all the regression analysis, p-values should also be provided.***
    *D)* ***Lines 287 – 298 – within each gene class, the genes were first sub-setted based on the number of ARE, and then the regression analysis was run. From the figures, it seems like regression was run with less than 10 genes. Why did the authors not run a multivariable regression analysis on all genes within the gene class and adjust for the number of ARE in the model? The slope/p-values of the multivariable regression should be compared with that univariate regression slope .***

We thank the reviewer for these suggestions. **A)** We prefer data representation in dot plot format which is more detailed (=including outliers) over a boxplot (=only representing mean + SD). **B)** We now included “The mean per group (red line) is indicated in A,C,E,G.” to clarify the meaning of the red lines. **C)** We also added the p-values for all the regression performed in figures 4/5/6. The p-values are indicated in the text, and the statistically significant (p<0.05) regression have been marked in figures with an “*”.

**D)** We agree with the reviewer that multivariable regression is a good alternative to our pipeline (univariate analysis). However, as also indicated by the reviewer, the limit in protein detection by MS prevents us from running accurate statistics on such low number of mRNA-protein pairs irrespective of analysis pipeline. Therefore, to clarify this limitation in data interpretation, we adjusted the results section accordingly and now state: “Of note, due to the low number of mRNA-protein pairs, these finding should be considered as mere indication for the regulatory effect of ARE clusters*.*” (line 374) and “Again, due to the low number of mRNA-protein pairs these findings should only be considered indicative*.*” (line 400). We believe that our revised statement better reflects the data shown in our paper.

16. **Inconsistent order of Figure labelling check Figures 3,4,5. Suggestion: Label top right panel as B and bottom left panel as C.**

We apologize for this inconsistency and have adjusted Figure 3 accordingly. Figure 4 and 5, however, could not be changed as the alteration would cause the figures to be called in the text in a disordered fashion.

17. **Figure 5B – What does n.s stand for?**

We now included ‘non-significant’ to the figure legend.

18. **Supplementary Figure 3 – since non-transformed values are plotted, the mean would be driven by extreme values. E.g., Supp Fig 3B (group >1).**

We thank the reviewer for this comment. We are in fact showing log10 transformed data for TPM and CN. We now adjusted the figure legend to better indicate the nature of the values: “(A-F) mRNA (TPM) and protein (CN) abundance in log10 space for…” (line 658).

19. **The authors should also mention in the discussion the role of *Trans*-acting DNA variants (eQTLs/pQTLs) may specifically affect mRNA or protein levels of genes which could also explain the low correlation seen.**

We thank the reviewer for the suggestion, and now state “… *has been attributed to differences in genetic variation, transcriptional rates, mRNA stability for mRNA abundance, and to protein synthesis and degradation rates for protein abundance…*”.
